# Abnormal circadian oscillation of hippocampal MAPK activity and power spectrums in NF1 mutant mice

**DOI:** 10.1186/s13041-017-0309-8

**Published:** 2017-07-03

**Authors:** Lei Chen, Tatiana Serdyuk, Beimeng Yang, Shuai Wang, Shiqing Chen, Xixia Chu, Xu Zhang, Jinjing Song, Hechen Bao, Chengbin Zhou, Xiang Wang, Shuangle Dong, Lulu Song, Fujun Chen, Guang He, Lin He, Ying Zhou, Weidong Li

**Affiliations:** Bio-X Institutes, Key Laboratory for the Genetics of Development and Neuropsychiatric Disorders (Ministry of Education), Shanghai Key Laboratory of Psychotic Disorders, and Brain Science and Technology Research Center, Shanghai Jiao Tong University, 800 Dongchuan Road, Shanghai, 200240 China

**Keywords:** *Nf1*^+/−^ mouse model, MAPK oscillation, Hippocampal rhythmic oscillations, Local field potentials, Spike activity

## Abstract

Studies have implied that the circadian oscillation of mitogen-activated protein kinase (MAPK) signal pathways is crucial for hippocampus-dependent memory. NF1 mouse models (*Nf1* heterozygous null mutants; *Nf1* ^+/−^) displayed enhanced MAPK activity in the hippocampus and resulted in memory deficits. We assumed a link between MAPK pathways and hippocampal rhythmic oscillations, which have never been explored in *Nf1* ^+/−^ mice. We demonstrated that the level of extracellular signal–regulated kinases 1 and 2 (ERK1/2) phosphorylation in *Nf1* ^+/−^ mice were significantly higher at nighttime than at daytime. Moreover, the in vivo recording revealed that for the *Nf1* ^+/−^ group, the power spectral density of theta rhythm significantly decreased and the firing rates of pyramidal neurons increased. Our results indicated that the hippocampal MAPK oscillation and theta rhythmic oscillations in *Nf1* ^+/−^ mice were disturbed and hinted about a possible mechanism for the brain dysfunction in *Nf1* ^+/−^ mice.

**Electronic supplementary material:**

The online version of this article (doi:10.1186/s13041-017-0309-8) contains supplementary material, which is available to authorized users.

## Background

Important transcriptional and translational events underlying long-term memory formation depend on the activation of mitogen-activated protein kinase (MAPK) signal pathways in the hippocampus [1–3]. Increased MAPK activity is the key pathophysiologic mechanism underlying neurofibromatosis type 1 (NF1) mutations in both mouse and humans [4]. NF1 is one of the most common single-gene causes of learning disabilities; studies on working memory and electrophysiology in NF1 mouse models (*Nf1* heterozygous null mutants; *Nf1* ^+/−^) have demonstrated that the NF1 mutation causes spatial learning disabilities and attention deficits [4, 5]. *Nf1* heterozygous null mutation results in enhanced ERK phosphorylation and increased gamma-aminobutyric acid (GABA) release in the hippocampus, which is reversed by the pharmacological downregulation of ERK signaling [6]. Past research has identified that lovastatin, a drug commonly used to treat hypercholesterolemia, could be a potent inhibitor of p21Ras/MAPK activity in the brain; in one study, lovastatin administration was found to decrease the levels of phosphorylated p44/42 MAPK in *Nf1* ^+/−^ mice [4]. In summary, abnormal elevation in MAPK activity is central to the pathophysiology associated with NF1 mouse models [7].

Evidence suggests that ERK1/2 MAPK phosphorylation (pERK1/2) in C57BL/6 mouse undergoes circadian oscillation in the hippocampus [8]; however, similar results have not yet been reported for other mouse lines. In addition, studies of multiple organisms have suggested that circadian rhythmicity is important for the formation, stability, and recall of memories [9]. Moreover, many studies have implied that the circadian oscillation of the MAPK signal pathway is critical for hippocampus-dependent memory [1–3] and that the oscillations of MAPK activity in the hippocampus may influence numerous processes, such as memory consolidation, neuronal survival, and ion channel activity [10–12]. However, the circadian cycle of the MAPK pathway in *Nf1* ^+/−^ mouse has not been illuminated, raising the question of whether hippocampal MAPK activity in *Nf1* ^+/−^ mouse models indicates any abnormal oscillations.

Previous studies have suggested a link between MAPK pathways and hippocampal rhythmic oscillations [13, 14]. Studies on rats have shown that theta-gamma comodulation accompanies memory retrieval in the hippocampus and that patterned brain stimulation may contribute to therapeutic strategies for cognitive disorders [15]. Specifically, a theta rhythm of 4–6 Hz, which is an overriding pattern in hippocampal circuits during some behaviors (e.g., information processing), is necessary for hippocampal-dependent spatial learning [16–21]. Recent studies have also reported that increased theta synchronization between the dorsal and ventral hippocampus may affect the cognitive process associated with the trace interval after a fear memory is retrieved successfully [22]. Nevertheless, the theta frequency spectrum is vital during periods of immobility with highly aroused states due to previously conditioned stimuli [16–18] or during time discrimination periods [23]. However, the mechanism by which the theta rhythm contributes to hippocampal functioning is still unknown. Furthermore, the hippocampus differentially operates the modifications to the theta frequency and its coupling during learning acquisition and retrieval states [15, 24, 25]. However, no study has investigated the hippocampal power spectrums in *Nf1* ^+/−^ mice, particularly the theta rhythmic oscillations.

Therefore, we hypothesized that the circadian oscillation of MAPK activity may influence the spatial learning and memory function of *Nf1* ^+/−^ mouse by affecting their hippocampal rhythmic oscillations. We examined the differences in an *Nf1* ^+/−^ mouse model during two periods (in daytime and nighttime) to identify the possible mechanisms in animal models of learning deficits.

## Methods

### Animals

Male *Nf1* ^+/−^ and wild-type (WT) mice (aged 12–16 weeks) were placed on a hybrid background of 129 T2/SvEmsJ-C57BL/6. The WT littermates were used as controls. The *Nf1* ^+/−^ mice were provided by the Alcino J. Silva Laboratory at the University of California, Los Angeles; the C57BL/6 mice were purchased from the Charles River Laboratories; and the 129 T2/SvEmsJ mice were purchased from the Jackson Laboratory. The mice had access to food and water ad libitum, except during testing times, and were maintained on a 12:12 h light:dark cycle (lighting time: 7:00 a.m.-7:00 p.m.). The mice were singly housed after surgery to prevent damage to the implanted electrode. All the experimental protocols were approved by the Institutional Animal Care and Use Committee of Shanghai Jiao Tong University.

### Antibodies

Rabbit anti-p44/42 MAPK (ERK1/2) (1:2000, Cell Signaling Technology, #9102), mouse anti-phospho-p44/42 MAPK (ERK1/2, Thr202/Tyr204) (1:2000, Cell Signaling Technology, #9101) were used.

### Western blotting

The mice were sacrificed and their hippocampal tissues were collected at two time periods (10:30–11:00 a.m. and 10:30–11:00 p.m.) [8, 26]. Hippocampal tissues from the WT littermates and *Nf1* ^+/−^, 129 T2/SvEmsJ, and C57BL/6 mice were collected and lysed in a radioimmunoprecipitation assay (RIPA) buffer (Sigma) that included a complete phosphatase inhibitor cocktail (Millipore, USA). A Bradford assay (Bio-Rad) was used to measure the protein concentration; the lysates (20 μg per lane) were separated using sodium dodecyl sulfate-polyacrylamide gel (12%) electrophoresis and transferred to the polyvinylidene fluoride (PVDF) membrane. The transferred membrane was blocked with 5% milk (BD, USA) for 1 h at room temperature, followed by an overnight incubation at 4 °C with a primary antibody. The membrane was incubated with horseradish peroxidase (HRP)–conjugated secondary antibody (Millipore, USA) for 1 h at room temperature. Immunoreactivity was detected with an enhanced chemiluminescence kit (Millipore, USA) and quantified using ImageJ software (NIH).

### Surgery and recording procedure for in vivo electrophysiology

**Fig. 1:**
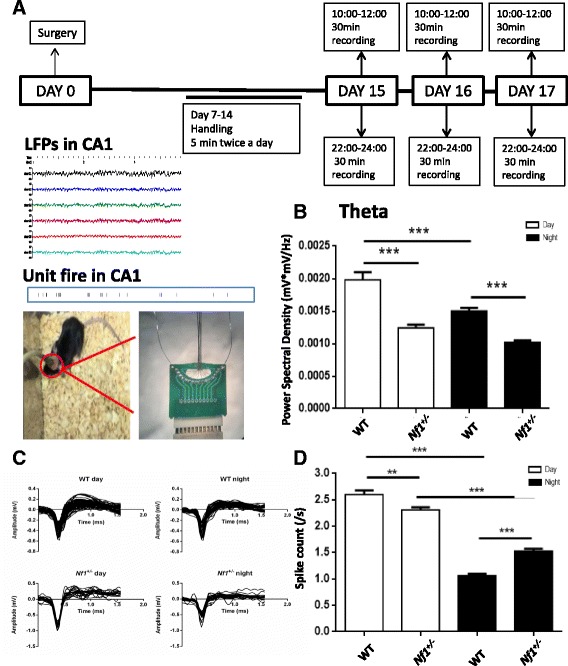
In vivo recording in CA1 demonstrates alterations in hippocampal rhythmic oscillations and firing rates in *Nf1* ^*+/−*^ mice. **a** The timeline of the in vivo recording experiments. The typical local field potentials (LFPs) recordings and unit spikes in CA1, the diagram of mice in recording with 16-channel microelectrode array were shown. **b** Histograms show the averaged power spectral density of the neuronal rhythmic oscillations (theta). Data are expressed as mean ± SEM (WT, *n* = 5; *Nf1* ^*+/−*^, *n* = 5). Two-way analysis of variance with repeated measures and post hoc Bonferroni tests was used to evaluate differences in local field potential power spectrum density in day and night recordings in *Nf1* ^*+/−*^ and WT groups. *** *p* < 0.001. **c** The differences in the spike waveforms of hippocampal pyramidal neurons between day and night in WT and *Nf1* ^*+/−*^ groups (WT, *N* = 5, n_day_ = 38, n_night_ = 37; *Nf1* ^*+/−*^, *N* = 5, n_day_ = 31, n_night_ = 25). **d** The spike firing rates of pyramidal cells. Comparison of the firing rates of pyramidal cells during day and night recordings (WT, *N* = 5, n_day_ = 38, n_night_ = 37; *Nf1* ^*+/−*^, *N* = 5, n_day_ = 31, n_night_ = 25). Paired t-test was used to evaluate differences in firing rates of pyramidal cells. **p < 0.01, ***p < 0.001

All surgeries were performed under stereotaxic guidance. The adult mice were anesthetized with sodium pentobarbital solution (10 mg/mL) for chronic implantations. The heads of the mice were placed securely in the stereotaxic frame (RWD Life Science, China). The 16-channel microelectrode array (16 tungsten wires with 80-μm tip diameter) were embedded in the left hemisphere with a dental cement mixture, and relevant coordinates were used to make extracellular recordings of local field potentials and record unit spikes (Fig. 1a). Stereotaxic coordinates for CA1 recordings (from bregma) were −1.94 mm AP, 1.25 mm ML, and 1.2 mm DV. The coordinates were determined using a mouse brain atlas [27]. Three stainless steel screws were fixed in the bone and one screw served as ground for the recordings. A reference electrode was placed over the parietal cortex or cerebellum. After surgery, the mouse’s health was monitored daily.

To record extracellular activity in vivo, we implanted a 16-channel microelectrode array with tungsten wires (80-μm tip diameter) in the hippocampus. The recordings were made 14 days after the surgery using a multichannel recording system (Fig. 1a) (extracellular single-cell unit activity and local field potentials (LFPs) in freely moving mice). The signals were first amplified by a 128-channel amplifier (Cerebus, Blackrock Microsystems, USA), with a filter frequency range of 0.3–5000 Hz, and visualized using a Cerebus 128-channel electrophysiology system (Blackrock Microsystems, USA). For 3 consecutive days, a series of 30-min recordings were made twice a day (10 am–12 pm and 10 pm–12 am) and visualized using the aforementioned system. LFPs and the neuron activity were analyzed using Offline Sorter (Plexon, USA), Neuroexplorer (Nex Technologies, USA), and Excel (Microsoft, USA) software. To analyze the multiunit activity of CA1 neurons, the probe channel in which unit activity could be seen visually to be located in the hippocampus was selected for multiunit detection. The signals were stored on a hard disk for offline analysis.

### Statistical analysis

*P* values <0.05 were considered statistically significant (*P < 0.05, ** *P* < 0.01, *** *P* < 0.001). All data were presented as means ± SEM and were analyzed using GraphPad Prism software. An unpaired two-tailed t test was used to measure the statistical differences between the two groups. A two-way ANOVA was used to compare the multiple groups’ data, followed by Bonferroni post hoc test. The electrophysiology data was analyzed using OriginPro 2015 (OriginLab Corporation, USA) and Student’s t tests or ANOVA.

## Results

### Alterations of hippocampal rhythmic oscillations and firing rates in *Nf1*^*+/−*^ mice

Studies have reported that NF1 patients are always with a wide range of neurological complications, including tumors, cognitive dysfunction, neuroimaging abnormalities and so on, and many of these complications may cause sleep disturbance [28, 29]. Since hippocampal rhythmic oscillations play important role in sleep and cognitive function and theta rhythm is necessary for hippocampal dependent spatial learning [30], to explore hippocampal theta rhythmic oscillations in *Nf1* ^+/−^ mice, we performed in vivo recording in CA1. To measure theta rhythmic alterations in the oscillatory activity of the WT and *Nf1* ^+/−^ groups, we recorded LFPs and spike activity from CA1 neurons at daytime and nighttime, respectively. The local field potential signal of the CA1 region was examined at daytime and nighttime from a microelectrode array. The changes in LFPs were quantified using power spectrums. The power spectral density of the theta frequency range (4–6 Hz) of the *Nf1* ^+/−^ mice significantly decreased at daytime and nighttime, compared with that of the WT group; moreover, the power spectral density of the theta frequency within the WT group decreased between daytime and nighttime. However, the power spectral density of the theta frequency of the *Nf1* ^+/−^ mice were the same at daytime and nighttime (Fig. 1b for theta frequency range N_WT_ = 5, N_*Nf1+/−*_ = 5; two-way ANOVA with repeated measures: row factor: F (63, 315) = 50.61, *P* < 0.0001, column factor: F (3, 15) = 33.81, *P* < 0.0001, interaction: F (189, 945) = 62.49; with Bonferroni post hoc test, *P* < 0.01; *n* = 6). The power spectral density of the alpha frequency range (7–12 Hz) decreased significantly in the *Nf1* ^+/−^ mice during daytime recordings, compared with the WT mice. However, no significant differences were observed between the WT and *Nf1* ^*+/−*^ groups in the alpha frequency range recorded at nighttime (Additional file 1: Figure S1). We observed that the power spectral density of the alpha frequency significantly decreased at daytime in the WT group (Additional file 1: Figure S1).

To further investigate the activity of CA1 neurons, we organized the multiple units’ activity signals into single unit spikes and then distinguished pyramidal neurons based on the widths of spike waveforms and shape of the waveforms. We sorted single unit spikes from the daytime and nighttime recordings of the multiple unit activity signals in the WT (*Nf1* ^+/−^) groups, from which we distinguished 38 (31) daytime and 37 (25) nighttime pyramidal neurons. The superimposed spike waveforms of the pyramidal neurons are shown in the Fig. 1c. The firing rates of the pyramidal neurons in the daytime and nighttime recording sessions were calculated. During the daytime recording sessions, the firing rates of pyramidal neurons in the *Nf1* ^+/−^ group decreased significantly (2.30 ± 0.57 spike/s), compared with that in the WT group (2.60 ± 0.85 spike/s) (Fig. 1d; paired t test: *P* = 0.0008, n_wt_ = 38, n _*Nf1*+/−_ = 31; data presented as mean ± SEM). During the nighttime recording session, the firing rates of pyramidal neurons in the *Nf1* ^+/−^ group increased significantly (1.53 ± 0.44 spike/s), compared with that in the WT group (1.06 ± 0.36 spike/s) (Fig. 1d; paired t test: *P* < 0.0001, n_wt_ = 37, n _*Nf1*+/−_ = 25; data presented as mean ± SEM). In the WT group, the firing rates of the pyramidal neurons decreased from 2.60 ± 0.85 spike/s during daytime recording sessions to 1.06 ± 0.36 spike/s during nighttime recording sessions (Fig. 1d; paired t test: *P* < 0.0001, n_wt day_ = 38, n _*WT* night_ = 37; data presented as mean ± SEM). In the *Nf1* ^*+/−*^ group, the firing rates of the pyramidal neurons decreased from 2.30 ± 0.57 spike/s during daytime recording sessions to 1.53 ± 0.44 spike/s during nighttime recording sessions (Fig. 1d; paired t test: *P* < 0.0001, n _*Nf1*+/− day_ = 31, n n _*Nf1*+/− night_ = 25; data presented as mean ± SEM).

### Disruption of MAPK activity oscillation in *Nf1*^*+/−*^ mice

**Fig. 2:**
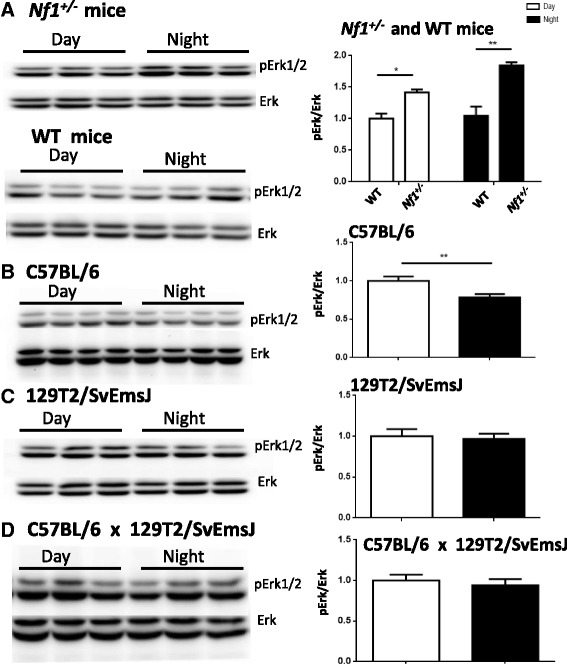
The level of P-MAPK activity of *Nf1* ^*+/−*^ mice in hippocampus shows different circadian oscillations compared with WT mice. **a** pErk1/2 expression in *Nf1* ^*+/−*^ mice and littermates WT mice were evaluated by western blot analysis at day and night. pErk1/2, normalized to Erk, the statistical quantification is shown in the right panel (*Nf1* ^*+/−*^ mice: *n* = 6 mice per time point, WT mice: *n* = 3 mice per time point, all groups normalized to WT in day), *p < 0.05, **p < 0.01. **b** pErk1/2 expression in C57BL/6 mice were evaluated by western blot analysis at day and night (10:30–11:00 a.m. and 10:30–11:00 p.m.). pErk1/2, normalized to Erk, the statistical quantification is shown in the right panel (*n* = 10 mice per time point, all groups normalized to C57BL/6in day), **p < 0.01. **c** pErk1/2 protein expression in 129 T2/SvEmsJ mice. pErk1/2 expression level, normalized to total Erk protein in the hippocampus (*n* = 7 mice per time point, all groups normalized to129T2/SvEmsJ in day). **d** pErk1/2 expression in mice with hybrid background of 129 T2/SvEmsJ-C57BL/6 mice were evaluated by western blot analysis at day and night. pErk1/2 expression level, normalized to total Erk protein in the hippocampus (*n* = 3 mice per time point, all groups normalized to mice in day)

Studies have indicated that hippocampal MAPK pathway and rhythmic oscillations have certain internal links [13, 14] and the behavior associated with theta frequency oscillations in hippocampal network contains a patterned activation of place cells in CA1, which have important effect on learning and memory [30]. In addition, researches have demonstrated that the MAPK activity in the hippocampus including CA1 region shows circadian oscillations [8]. To explore the reason of the disturbed hippocampal oscillations in *Nf1* ^+/−^ mice, western blotting tests were performed to detect the oscillation of hippocampal MAPK activity. The mice were sacrificed and their hippocampal tissues were collected at two time periods (10:30–11:00 a.m. and 10:30–11:00 p.m.), when the ERK phosphorylation show highest and lowest level [8, 26]. The Western blot analysis revealed a pronounced difference in pERK1/2 levels between the *Nf1* ^+/−^ mice and WT littermates. The pERK1/2 in NF1 knockout (heterozygous KO) mice were significantly higher at nighttime than at daytime (Fig. 2a; unpaired two-tailed t test: *Nf1* ^+/−^ daytime *n* = 6, *Nf1* ^+/−^ nighttime *n* = 6, *t* = 5.947, *p* < 0.01). We did not observe any differences in the pERK1/2 activity of WT mice between the two time periods (Fig. 2a; unpaired two-tailed t test: WT daytime *n* = 3, WT nighttime *n* = 3, *t* = 0.8581, *p* > 0.05). The *Nf1* ^+/−^ mouse model showed abnormally higher MAPK activity, compared with the WT mice, both at daytime and nighttime, verifying that this mouse model presented an aberrant level of RAS-MAPK pathway (Fig. 2a; two-way ANOVA: row factor: F(1,20) = 6.727, *p* = 0.0174; column factor: F(1,20) = 44.69, *p* < 0.01; interaction: F(1,20) = 4.387, *p* = 0.0491). Because of the special background of the *Nf1* ^+/−^ mice (hybrid background of 129 T2/SvEmsJ-C57BL/6), we also detected ERK activity in the C57BL/6 and 129 T2 mice. Consistent with previous studies, the C57BL/6 mice had evident circadian oscillations during ERK activity [8]; however, 129 T2 mice had no oscillations (Fig. 2b c), and the hybrid mice bred by C57BL/6 and 129 T2 also showed no circadian oscillations in MAPK activity (Fig. 2d), indicating that the loss of MAPK oscillation in WT littermates may be caused by the hybridized background. These results indicated that the oscillation of MAPK activity in *Nf1* ^+/−^ mice were disturbed, compared with that in WT littermates.

## Discussion

To explore alterations in oscillatory activity in the *Nf1* ^+/−^ and WT groups, we recorded LFPs and spike activity in CA1 neurons at daytime and nighttime, respectively. The results of in vivo recording demonstrate the abnormal alterations in hippocampal theta rhythmic oscillations and firing rates in the *Nf1* ^+/−^ mice. In addition, the power spectra density of the theta frequency range significantly decreased at daytime and nighttime in the *Nf1* ^+/−^ group; this group also exhibited overexpressed MAPK activity at nighttime. After sorting the multiple units’ activity signals into single unit spikes, we distinguished pyramidal neurons based on the widths of spike waveforms and shape of the waveforms. During the daytime recording sessions, the firing rates of pyramidal neurons in the *Nf1* ^+/−^ mice decreased compared with those of their WT littermates, whereas during nighttime recording sessions, the firing rates of pyramidal neurons increased significantly. These electrophysiology findings prove the unusual alterations in LFP and spike activity in *Nf1* ^+/−^ mice. Considering that the theta rhythm is a main pattern in hippocampal circuits and is necessary for hippocampal-dependent learning [16–18], we inferred that the abnormal theta rhythm in *Nf1* ^+/−^ mice may be a neuronal basis of the dysfunction in cognitive behavior. Several previous studies have reported that the hyperpolarization-activated cyclic nucleotide-gated (HCN) channels have an important role in regulating theta cycle in hippocampal circuits [31]. Moreover, HCN channels are regulated by serine/threonine kinase, p38-mitogen-activated protein (MAP) kinase, belonging to the MAPK family [32, 33]. In this study, we identified a link between MAPK pathways and hippocampal theta rhythm. The theta frequency oscillation may be regulated by MAPK signal pathways by affecting the function of HCN channels during the circadian cycle, which is needed to be further studied in *Nf1* ^+/−^ mouse model. According to reviewer’s suggestion, we added: Spikes and firing rate in neuron play important role in information transmissions among the brain, which are critical in cognitive function [34]. Studies have shown that the MAPK signaling cascade has critical roles in regulation of neuronal excitability [35], and prior studies indicate that, for specific patterns of stimulation, MAPK may function in the regulation of neuronal excitability in hippocampal area CA1 [36]. Moreover, the progressive increase in spiking observed during theta-burst stimulation (TBS) represents a form of physiologic temporal integration that is dependent on ERK MAPK activity [36]. In this study, the abnormal alterations in spike activity in *Nf1* ^+/−^ mice may be caused by the unusual MAPK oscillation activity in this mouse model, further research should be performed about the specific links between pERK1/2 and neuronal firing in *Nf1* ^+/−^ mouse model.

Numerous studies have investigated rhythmicity in central nervous system tissues, including those on memory processing and cognition. Recent studies have demonstrated that the oscillation of hippocampal MAPK activity influences cognitive function. Evidence suggests that pERK1/2 undergoes a circadian oscillation in the hippocampus [8]. Both the MAPK and cyclic adenosine monophosphate (cAMP) signal pathways have important roles in the consolidation of hippocampus-dependent memory [37]. In addition, the circadian oscillation of pERK1/2 is accompanied by the changes in cAMP response element–binding protein (CREB) activity [8]. The persistence of long-term memories may depend on the reactivation of cAMP/MAPK/CREB transcriptional signal pathway in the hippocampus during a circadian cycle [8, 38]. Moreover, *Bmal1* ^−/−^ mice have no diurnal change in cAMP and MAPK activity, indicating defects in learning and spatial memory, impaired LTP, and disordered contextual fear memory [36–38]. In addition, a previous study reported that levels of phosphorylation MAPK in the chick pineal gland exhibited circadian rhythms, suggesting that components in the Ras-MAPK pathway are activated in a circadian manner [39, 40]. Studies of *Drosophila* have identified that null mutations of the NF1 produce abnormalities of circadian rhythms in locomotor activity [41–43]. Substantial evidence indicates that the oscillation of MAPK activity is important for the mechanisms of learning and memory. The level of ERK1/2 phosphorylation in the NF1 heterozygous KO mice was significantly higher at nighttime than at daytime. However, we did not observe any difference in pERK1/2 activity of the WT mice between the two time periods. We found oscillations of MAPK activity are abnormal in *Nf1* ^+/−^ mice for the first time. In addition, we also found WT mice in day showed the maximum power spectral density of the theta frequency, but the mice demonstrated the lowest pERK1/2 level in daytime (Additional file 2: Figure S2).While, the heterozygous KO mice showed the minimum power spectral density in theta frequency and the highest pERK1/2 activity in nighttime(Additional file 2: Figure S2). It seems like that there may be certain correlation between theta oscillation and MAPK level in hippocampus (Additional file 2: Figure S2). Considering that hippocampus-dependent memories are regulated by MAPK activity oscillation [42, 43], our data suggest that the circadian oscillation of MAPK activity may be one of reasons which cause the cognitive defects in *Nf1* ^+/−^ mice. Furthermore, previous studies have demonstrated elevated p-MAPK activity in animal models of NF1 result in cognitive deficits [4, 8], the Western blotting data verified this result.

The results of this study firstly verify the aberrant hippocampal MAPK oscillation and power spectrum rhythm in the *Nf1* ^+/−^ mouse model. However, the molecular mechanisms underlying the abnormal MAPK circadian oscillation and whether the aberrant MAPK activity in oscillation may lead to a variance in spatial learning and memory remain unclear, and the relationship between the hippocampal MAPK activity, particularly the ERK, and power spectrum rhythm, including theta frequency, warrants further investigation.

## Conclusions

This study demonstrated that both the oscillation of MAPK activity and power spectrum rhythm of the *Nf1* ^+/−^ mice were disturbed in comparison with that of their WT littermates; these results elucidated certain internal relations between MAPK pathways and theta frequency oscillation, which have noticeable effect for further mechanism exploring in the *Nf1* ^+/−^ mouse model.

## Additional files

Additional file 1: Figure S1. The correlation is shown between theta oscillation and MAPK level in hippocampus. The averaged power spectral density of the neuronal rhythmic oscillations (theta) was shown. The pErk1/2 expression in *Nf1* ^*+/−*^ mice and littermates WT mice were evaluated by western blot analysis at day and night. (PDF 421 kb)

Additional file 2: Figure S2. In vivo recording in CA1 demonstrates alterations in hippocampal rhythmic oscillations and firing rates in *Nf1* ^*+/−*^ mice. **a** The local field potentials (LFPs) recordings in CA1(WT mice). First trace- unfiltered LFPs, second trace- alpha oscillations (filtered 7–12 Hz). **b** Histograms show the averaged power spectral density of the neuronal rhythmic oscillations (alpha). Data are expressed as mean ± SEM (WT, *n* = 5; *Nf1* ^*+/−*^, *n* = 5). Two-way analysis of variance with repeated measures and post hoc Bonferroni tests was used to evaluate differences in local field potential power spectrum density in day and night recordings in *Nf1* ^*+/−*^ and WT groups. ***p < 0.001. (PDF 212 kb)

Additional file 3: Supplementary tables were shown as the spike firing rates of pyramidal cells in mice. (ZIP 106 kb)

## Abbreviations

AP: Anteroposterior
cAMP: Cyclic adenosine monophosphate
CREB: CAMP response element–binding protein activity
DV: Dorsoventral
ERK1/2: Extracellular signal–regulated kinases 1 and 2
GABA: Gamma-aminobutyric acid
HCN: Hyperpolarization-activated cyclic nucleotide-gated
LFPs: Local field potentials
MAPK: Mitogen-activated protein kinase
ML: Mediolateral
NF1: Neurofibromatosis type 1
pERK1/2: ERK1/2 phosphorylation
PVDF: Polyvinylidene fluoride
RIPA: Radioimmunoprecipitation assay
