# Supplementary figures and images for: Abnormal circadian oscillation of hippocampal MAPK activity and power spectrums in NF1 mutant mice
Source: Mol Brain. 2017 Jul 3;10:29. doi: 10.1186/s13041-017-0309-8 (PMC5496334; doi:10.1186/s13041-017-0309-8)

# Theta

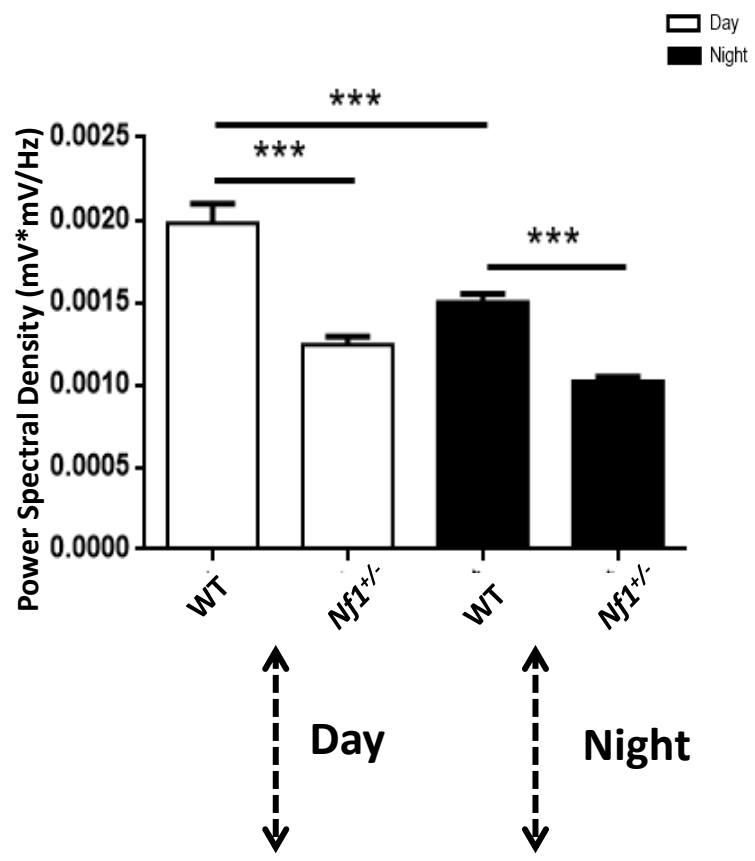

## *Nf1*<sup>+/-</sup> and WT mice

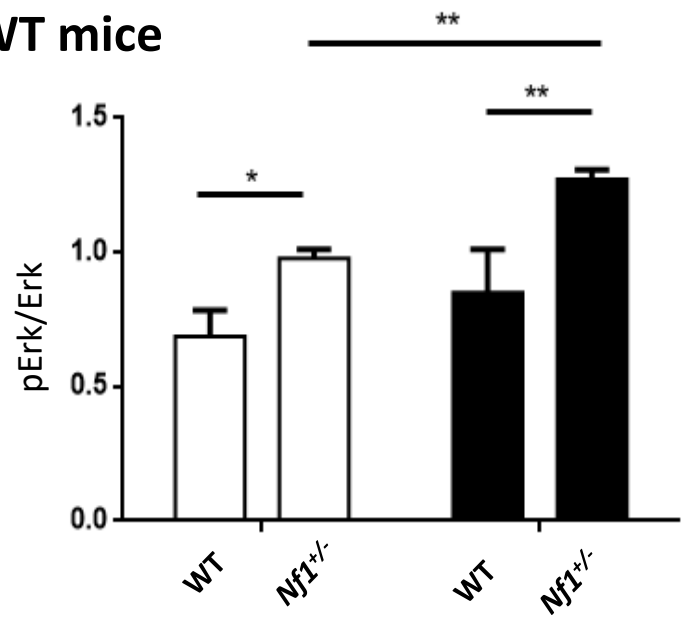

Supplement: Supplementary file 1 — The correlation is shown between theta oscillation and MAPK level in hippocampus. The averaged power spectral density of the neuronal rhythmic oscillations (theta) was shown. The pErk1/2 expression in Nf1 +/− mice and littermates WT mice were evaluated by western blot analysis at day and night. (PDF 421 kb) [file 13041_2017_309_MOESM1_ESM.pdf]

## A. LFPs and Alpha oscillations in CA1

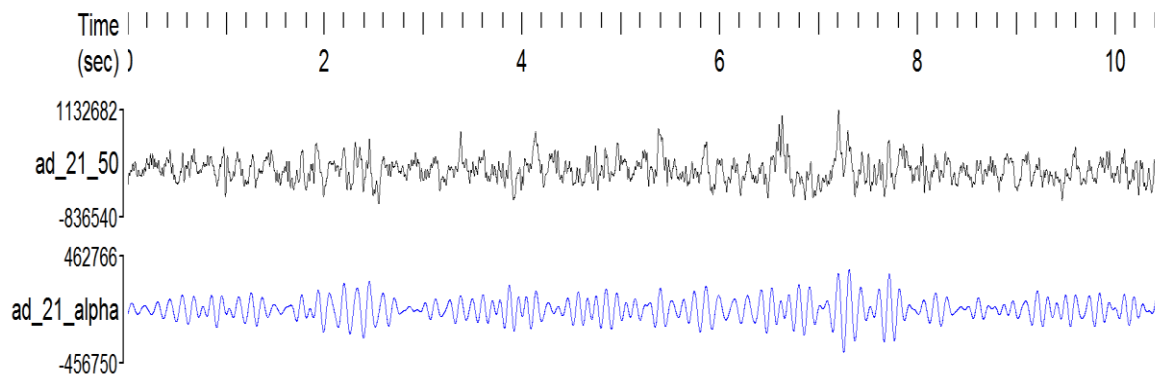

## B. Alpha

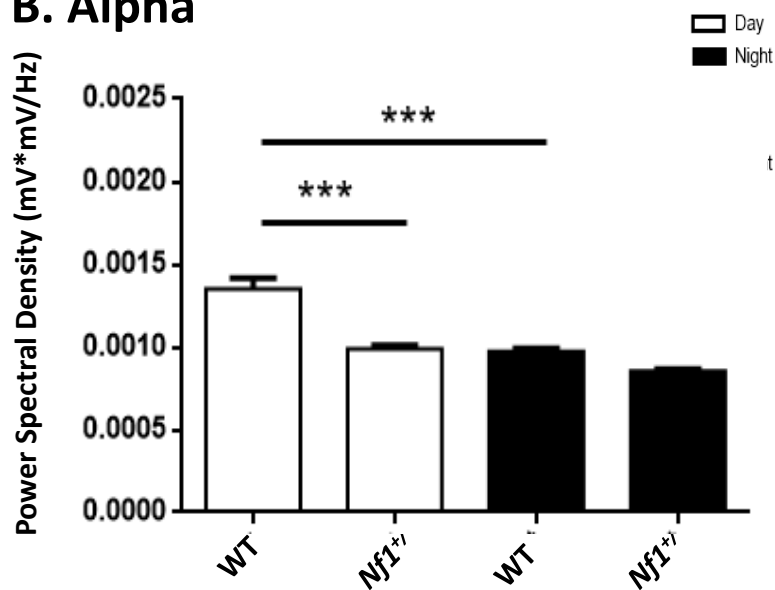

Supplement: Supplementary file 2 — In vivo recording in CA1 demonstrates alterations in hippocampal rhythmic oscillations and firing rates in Nf1 +/− mice. a The local field potentials (LFPs) recordings in CA1(WT mice). First trace- unfiltered LFPs, second trace- alpha oscillations (filtered 7–12 Hz). b Histograms show the averaged power spectral density of the neuronal rhythmic oscillations (alpha). Data are expressed as mean ± SEM (WT, n = 5; Nf1 +/−, n = 5). Two-way analysis of variance with repeated measures and post hoc Bonferroni tests was used to evaluate differences in local field potential power spectrum density in day and night recordings in Nf1 +/− and WT groups. ***p < 0.001. (PDF 212 kb) [file 13041_2017_309_MOESM2_ESM.pdf]
