# Supplementary material for: Abnormal circadian oscillation of hippocampal MAPK activity and power spectrums in NF1 mutant mice
Source: Mol Brain. 2017 Jul 3;10:29. doi: 10.1186/s13041-017-0309-8 (PMC5496334; doi:10.1186/s13041-017-0309-8)
Supplement: Supplementary file 3 — Supplementary tables were shown as the spike firing rates of pyramidal cells in mice. (ZIP 106 kb) [file 13041_2017_309_MOESM3_ESM.zip › Supplementary information file 2.pdf]

Table1

Sample	Protein level	
	pErk1/2	Erk
D-KO-1	5137.719	7042.619
D-KO-2	5366.426	7407.912
D-KO-3	4671.305	6658.205
D-KO-4	10796.88	12991.39
D-KO-5	9733.004	12893.1
D-KO-6	8457.418	13026.1
N-KO-1	6924.426	6492.083
N-KO-2	6532.841	6772.912
N-KO-3	5387.134	5716.79
N-KO-4	8736.296	9008.56
N-KO-5	10253.59	11397.68
N-KO-6	11711.88	13518.15
D-WT-1	3794.719	5752.255
D-WT-2	2680.598	5829.79
D-WT-3	2485.305	5774.79
N-WT-1	2522.598	5592.497
N-WT-2	2967.305	4942.669
N-WT-3	4322.426	5020.497

Table2

Sample	Protein level	
	pErk1/2	Erk
D-B6-1	9096.075	8695.125
D-B6-2	7183.246	7854.125
D-B6-3	6051.539	7601.125
D-B6-4	8324.66	10094.71
D-B6-5	8124.953	10444.13
D-B6-6	7974.075	11340.83
D-B6-7	8509.66	11763.83
D-B6-8	9596.631	16231.95
D-B6-9	8712.045	13756.41
D-B6-10	9593.752	14245.87
N-B6-1	4678.296	8304.125
N-B6-2	5523.832	8896.418
N-B6-3	7040.246	9196.711
N-B6-4	6457.539	8709.418
N-B6-5	5235.004	8371.418
N-B6-6	6334.004	9611.832
N-B6-7	8325.752	13662.17
N-B6-8	6422.681	12754.87
N-B6-9	7045.217	14707.29
N-B6-10	6088.095	13360.87
N-B6-9	7045.217	14707.29
N-B6-10	6088.095	13360.87

Table3

Sample	Protein level	
	pErk1/2	Erk
D-129-1	6162.255	6477.497
D-129-2	6642.548	7357.79
D-129-3	6292.548	7534.083
D-129-4	7393.246	12453.51
D-129-5	6768.953	12231.34
D-129-6	6166.125	10691.39
D-129-7	7300.246	10598.68
N-129-1	6293.548	6729.497
N-129-2	6063.548	7542.497
N-129-3	4862.255	7525.912
N-129-4	6275.832	9421.267
N-129-5	6096.418	9303.267
N-129-6	5673.296	9431.439
N-129-7	5770.175	9203.024

Table4

Sample	Protein level	
	pErk1/2	Erk
D-B6x129-1	6729.861	8297.569
D-B6x129-2	7440.276	7944.569
D-B6x129-3	5702.569	7735.276
N-B6x129-1	5021.861	7662.276
N-B6x129-2	6267.861	7624.276
N-B6x129-3	6095.276	7079.154

Supplementary information 2: Table1-4 were shown as western blotting raw data in figure2a-d respectively. Immunoreactivity was quantified using ImageJ software (NIH), each value represents the grayscale, which was used to quantify the protein level (D: day; N: night; KO: NF1 KO mcie; B6: C57BL/6 mice; 129: 129T2/SvEmsJ mice; B6x129: mice with hybrid background of 129T2/SvEmsJ-C57BL/6).
